# De novo 1Mb interstitial deletion of 8p22 in a patient with slight mental retardation and speech delay

**DOI:** 10.1186/1755-8166-7-25

**Published:** 2014-04-15

**Authors:** Giovanna Piovani, Giulia Savio, Michele Traversa, Alba Pilotta, Giuseppina De Petro, Sergio Barlati, Chiara Magri

**Affiliations:** 1 Biology and Genetics Division, Department of Molecular and Translational Medicine, University of Brescia, Viale Europa 11, 25123 Brescia, Italy; 2 Centro di Auxoendocrinologia, Department of Paediatrics, Spedali Civili, Brescia, Italy

**Keywords:** SNP Array, Deletion, 8p22, Developmental delay, Hypotonia, TUSC3, miR-383, SGCZ

## Abstract

We report on a nine years old girl born after 41 weeks of normal gestation with psychomotor retardation, speech delay and minimal dysmorphic signs: antimongolic cut eyes, small mouth, short philtrum and hypertelorism.

The use of the high-resolution Affymetrix Human Mapping GeneChip 250 K NspI array allowed the characterization of a *de novo* 1Mb deletion on the short arm (p22) of a chromosome 8. Molecular cytogenetic-FISH with BAC probes (RP11) confirmed the deletion. The deleted region includes part of the sarcoglycan zeta (SGCZ) gene, involved in the sarcoglycan complex formation, and the microRNA 383. The deletion described in our patient falls 319 Kb upstream of the Tumor Suppressor Candidate 3 (TUSC3) gene. In this chromosomal region, a limited number of cases of overlapping deletions, of variable extensions and characterized by heterogeneous clinical phenotype, have been reported. The deleted region described in our patient is the smallest among those so far described in this region.

## Background

Chromosomal aberrations are considered to be the most frequent cause of unexplained developmental delay (DD), intellectual disability (ID) and multiple congenital anomalies (MCA). Emerging and increasing availability and sensitivity of the technologies, new molecular cytogenetic, array-based whole genome screening, showed that the contribution of chromosomal aberrations in ID is not limited to the extent evidenced by karyotyping alone (6–10%) but can be increased to as high as 20–30% using the mentioned methods [1]. Interstitial microdeletion and microduplication syndromes account for 50–60% of the total submicroscopic chromosomal abnormalities detected in ID/MCA patients. In the course of a systematic screening of patients with unexplained ID, by the use of the high-resolution SNP-array, we have identified, in a 9 years-old patient affected by mild ID, a *de novo* 1Mb interstitial deletion in the 8p22.3 chromosomal region overlapping SGCZ gene, miR-383 and mapping upstream of the TUSC3 gene.

The discovery of new patients with microdeletions in 8p22 will lead to a better understanding of the roles that the deleted genes play in the clinical outcome and will facilitate both comprehensive medical care and accurate recurrence risk assessment for the family.

## Case presentation

The proband is the second female child of non consanguineous Caucasian parents. Both her parents and her sister are healthy and familiar history is negative for malformation or developmental delay.

The patient was delivered uneventfully at 41 weeks of gestation after a normal pregnancy without intrauterine infection. No prenatal diagnosis was performed.

At birth, the weight was 3,470 g (50^th^ centile) and length was 50 cm (50^th^ centile); in subsequent evaluations she showed regular stature-weight growth and slight delay in the acquisition of motor milestones. She demonstrated episodes of bronchiolitis and ocular examinations revealed congenital strabismus and hypotonia with minimal dysmorphic signs. These were antimongolic cut eyes, small mouth, short philtrum, hypertelorism, hands with long and tapered fingers, feet with long toes, valgus knee and only one groove of the right hand. At the age of six years, when admitted to the school, she showed evident psychomotor retardation and speech delay. Reduced muscle tone was associated with distal ligamentous laxity, and preserved diadococinesia, hindrance in distal mobility as reported in coordination exercises and fingers singolarization.

Brain MRI showed presence of moderate signal hyperintensity in the long TR sequences involving posterirly the periventricular white matter of the radiated crowns in a symmetrical way, being of uncertain significance.

There was a delay in language capacity primarily on the expressive side. Simple orders understanding capability was preserved but difficulty in comprehension orders has been recorded. Spontaneous words were poorly understood, and moderate hindrance in the execution of praxis bucco-lingual.

The general development quotient (Griffiths score) was below the normal value, 64, with discordant trend; decrease in hearing-language score, (53.8), and hand-eye coordination, (59), was recorded. The global evaluation of cognitive performances revealed a low grade of mental retardation requiring psycho-pedagogical help.

At the last examination at 8 years of age, the clinical picture was unchanged.

### Results

#### Affymetrix mapping human 250 K NspI array and copy-number analysis

**Figure 1:**
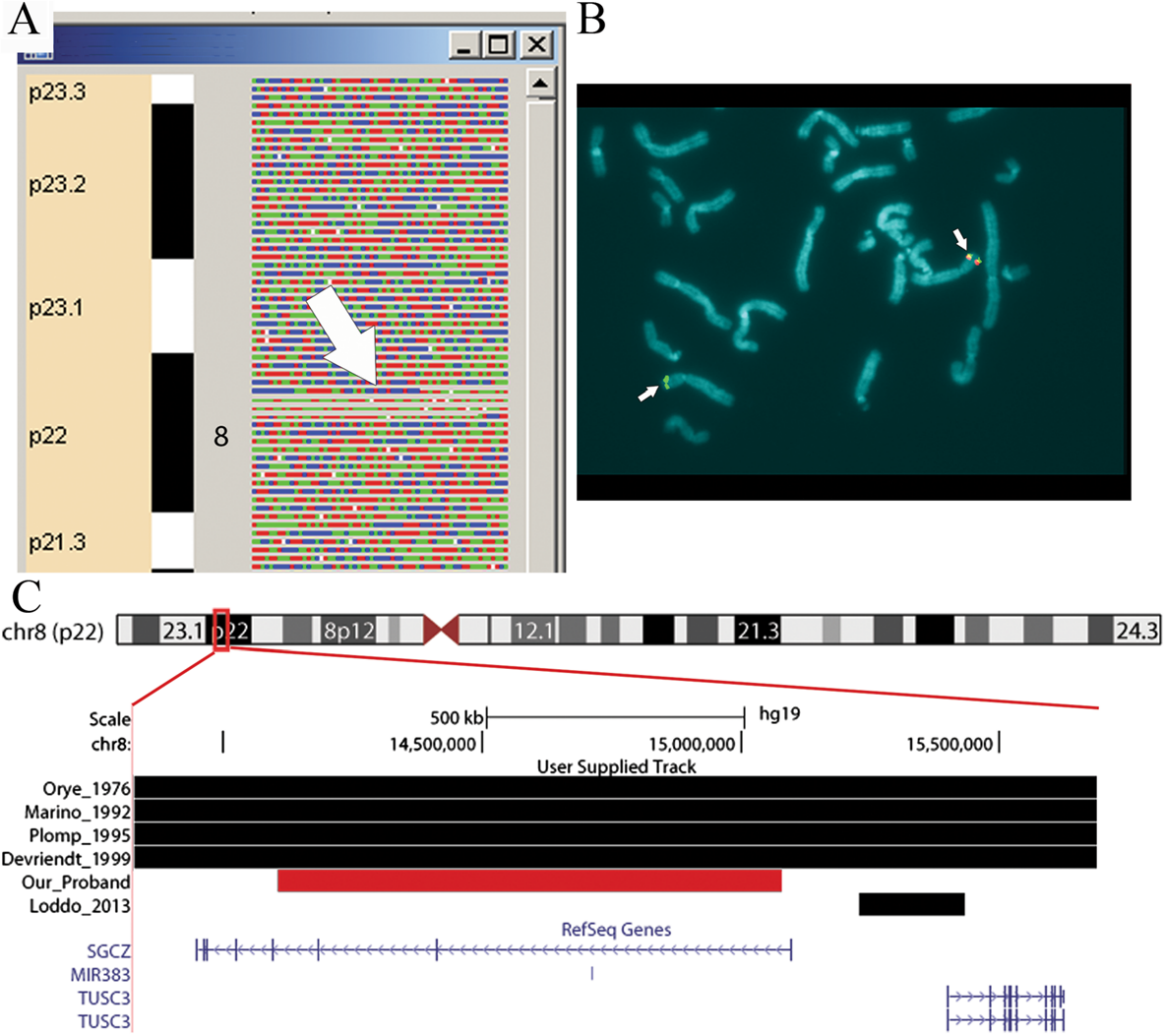
**Visualisation of the 8p22 interstitial deletion. A** Genotype and CN state visualisation of SNPs on short arm of chromosome 8 with GenotypeColour. The column corresponding to the genotype profile of the proband is made of many squares symbolizing SNPs; the colour of the squares depends on the SNP genotype, whereas their height is proportional to their CN states. See Barlati et al. [17] for references. In the proband, a partial deletion of chromosome 8, characterized by the absence of heterozygous genotypes and squares of reduced height, is indicated by the white arrow. **B** FISH analysis on metaphase spread derived from the proband. Signals derived from BAC clones RP11-21H13 and RP11-399 J23 (control probe) are in red and green, respectively. The chromosome 8 with the deletion displayed only the signal for the RP11-399 J23 control probe. **C** UCSC Genome browser image of the 8p23.1-p21.3 region. The deletion described in our patient (red bar) is displayed together with those reported by [3-6]. The genes annotated in this region are reported at the bottom of the figure.

The SNP-array analysis evidenced in our patient a *de novo* interstitial deletion of 1Mb at 8p22. The proximal breakpoint mapped between 14,089,730 bp (SNP_A-2290138 last probe not deleted) and 14,105,921 bp (SNP_A-1992355 first deleted probe); while the distal breakpoint was located between 15,078,161 bp (SNP_A-2133346) and 15,092,243 bp (SNP_A-2103758) (last probe deleted and first not deleted, respectively) (Figure 1A).

#### Cytogenetic and FISH analysis

Chromosome preparations revealed a normal, (46,XX), female karyotype. The BAC clones, RP11-21H13 and RP11-399 J23, used in FISH experiments, confirmed the deletion (Figure 1B).

Conventional chromosomal and FISH analysis, with the same probes, showed absence of the deletion in the parents.

The bioinformatic analysis of the sequences involved in the chromosomal rearrangements was carried out using the University of California Santa Cruz (UCSC) Genome Browser [2] on the NCBI build GRCh37 of the human genome.

#### Sequencing of the TUSC3 gene

The sequencing of the coding region of the TUSC3 gene did not highlight the presence of deleterious functional variants.

### Discussion

We report a 1Mb interstitial deletion in the 8p22 locus identified in a girl with psychomotor and speech delay. Standard cytogenetic analysis resulted in a normal karyotype and only subsequent analysis by SNP array revealed a deletion in the short arm of one chromosome 8.

In literature, deletions of this region are reported in a small number of cases [3-6]. In the majority of cases, the deletions spam from band p22 to the subtelomeric region and include, therefore, a large part of the short arm of chromosome 8. The carriers of these deletions show heterogeneous clinical phenotypes ranging from mental retardation with minimal dysmorphic signs [6] to important mental retardation [3] and dysmorphic signs like narrow cranium, high forehead, epicanthic folds, flat nasal bridge, low-set but normal ears, retrognathy, short neck and broad chest with wide-set nipples [5]. In addition, in some patients, the deletion of chromosome 8p has been found associated also with congenital heart malformations [3,4]. Our patient, with low mental retardation, presents neither congenital heart malformations nor the major dysmorphic signs described in other patients. The heterogeneity and the dissimilarities of the observed clinical phenotypes are mainly due to the differences in the size of the deletions and in their different percentage of overlapping. In the earliest reports, the extent of the aberrations was detected only by standard cytogenetic analysis therefore a molecular characterization of old cases would be desirable to perform better genotype to phenotype correlations. To date our deletion highlighted with new techniques is one of the smallest so far described in the 8p22 locus.

The observed *de novo* 8p22 deletion includes 3 out of the 8 exons of the sarcoglycan zeta gene (SGCZ) and the entire microRNA (miRNA) 383 (Figure 1C). The SGCZ gene encodes for the ζ-sarcoglycan, one of the six proteins (α, β, γ, δ, ϵ, ζ) constituting the sarcoglycan complex (SGC) that provides a mechanosignaling connection from the cytoskeleton to the extracellular matrix. Mutations in the sarcoglycan genes α-, β-, γ- and δ (in particular deletions of exons), lead to a group of autosomal recessive disorders defined limb girdle muscular dystrophies (LGMD). Up to now, no neuromuscular disorders have been correlated with mutations or partial deletions of the ζ subunit gene. In our patient, no clinical sign overlapping those typical of the LGMDs was observed. This suggests that if the SGCZ gene is involved in the LGMD phenotype, as for the other sarcoglycan genes, two hits are required for the onset of the disease. However, since no neuromuscular disorders have been so far correlated with the ζ subunit, we cannot exclude that CNVs in this gene are neutral or responsible of a complete different phenotype from that of dystrophies.

The DNA sequence inside intron 3 of SGCZ gene encodes also for miR-383. miRNAs are short endogenous, single-stranded RNAs that exert transcriptional and post-transcriptional regulation of gene expression. Altered miRNA expression levels have been found in the brains of patients affected by ID, neurological and psychiatric diseases, suggesting that their dysregulation may contribute to these clinical phenotypes [7,8]. However in literature no direct evidences are reported showing that deletions of miR-383 impair neurodevelopment, but evidences of its involvement in tumorigenesis are present. Actually, a recent study demonstrated that miR-383 is an oncosuppressor gene that directly targets Cyclin D1 and then inhibits its downstream effectors. The authors also demonstrated that a decreased expression of miR-383 can deregulate the cyclin-dependent kinase 4 expression and promote DNA damage and the onset of testicular embryonal carcinoma [9]. Therefore we cannot exclude that the deletion described in our patient might predispose to tumour development, and a follow up of the patient in the future years would be advisable.

The deletion described in our patient falls 319 Kb upstream of the Tumor Suppressor Candidate 3 (TUSC3) gene (Figure 1C) encoding a subunit of the ER-bound oligosaccharyltransferase (OST) complex that catalyzes a pivotal step in the protein N-glycosylation process [10]. Homozygous mutations and deletions inside this gene have been described both in patients with nonsyndromic autosomal recessive form of mental retardation (MIM #611093) [10-14] and in a patient with few dysmorphic features and a syndromic ID [15]. Heterozygous mutations and deletions of TUSC3, instead, have been reported in healthy subjects. The phenotype of our patient (moderate mental retardation and only mild dysmorphic traits) is compatible with that of impairment of TUSC3 gene; but the sequencing of the coding region of the gene did not highlight the presence of deleterious functional variants that in combination with the deletion upstream of the gene could point to the involvement of TUSC3 in the pathological phenotype. We cannot exclude, however, the presence of mutations in regulatory elements that could alter the transcription level of the gene.

## Conclusions

In conclusion our patient with slight mental retardation and speech delay carries a *de novo* 1Mb deletion never observed before in healthy subjects that could be responsible of the clinical phenotype and that could also represent a risk factor for cancer development in adulthood. Only the discovery of others patients with microdeletions overlapping to that described in our patient will lead to a better understanding of the roles of the deleted genes in the clinical outcome.

### Materials and methods

#### Affymetrix mapping human 250 K NspI array and copy-number analysis

The DNA of the proband was analysed with the Affymetrix GeneChip Human Mapping 250 K NspI arrays and processed according to the instructions provided in the Affymetrix GeneChip Human Mapping 500 K Assay Manual. Initial analysis and quality assessment of the array data were performed using GTYPE 4.1. Copy number state determination was performed with the Affymetrix CNAT4 software. The copy number state determination analysis was performed by comparing our sample with a reference set of 48 Hapmap samples available on the Affymetrix web site [16]. The genotype and copy number state of the patient genome were visualized with GenotypeColour software [17].

#### Cytogenetic and FISH analysis

Chromosome preparations were obtained on cultured pheripheral lymphocytes and performed according with standard cytogenetic protocol using QFQ banding at 450 bands resolution. The BAC clones, used in FISH experiments, were provided by CHORI [18]. Fluorescence *in situ* hybridisation (FISH) was performed as described previously [19].

#### Sequencing of the TUSC3 gene

The exons of the TUSC3 gene were sequenced on an ABI PRISM 3100 sequencer (Applied Biosystems, Foster City, CA) according to standard methods. Primers sequences and sequence conditions are available upon request.

## Consent

Written informed consent was obtained from the patient’s parent for publication of this Case report and any accompanying images. A copy of the written consent is available for review by the Editor-in-Chief of this journal.

## Competing interests

The authors declare they have no competing interests.

## Authors’ contributions

GP and CM designed the study, drafted the paper and gave the final approval of the manuscript. GP was responsible for the conventional and molecular cytogenetic analysis. CM and SB interpreted the data of SNP-array. GS and MT performed SNP-array and sequencing. AP and GDP contributed to revising the paper. All authors read and approved the final manuscript.
